# Involvement of Dynamin-Related Protein 1 in Free Fatty Acid-Induced INS-1-Derived Cell Apoptosis

**DOI:** 10.1371/journal.pone.0049258

**Published:** 2012-11-14

**Authors:** Liang Peng, Xiuli Men, Wenjian Zhang, Haiyan Wang, Shiqing Xu, Qing Fang, Honglin Liu, Wenying Yang, Jinning Lou

**Affiliations:** 1 Institute of Clinical Medical Sciences, China-Japan Friendship Hospital, Beijing, China; 2 Department of Pathophysiology, North China Coal Medical University, Tangshan, China; 3 Department of Cell Physiology and Metabolism, University of Geneva Medical School, Geneva, Switzerland; University of Bremen, Germany

## Abstract

Elevated extracellular free fatty acids (FFAs) can induce pancreatic beta cell apoptosis, thereby contributing to the pathogenesis of type 2 diabetes mellitus (T2D). Mitochondrial dysfunction has been implicated in FFA-induced beta cell apoptosis. However, molecular mechanisms linking mitochondrial dysfunction and FFA-induced beta cell apoptosis are not clear. Dynamin-related protein 1 (DRP-1) is a mitochondrial fission modulator. In this study, we investigated its role in FFA-induced INS-1 beta cell apoptosis. DRP-1 protein was promptly induced in INS-1 cells and rat islets after stimulation by FFAs, and this DRP-1 upregulation was accompanied by increased INS-1 cell apoptosis. Induction of DRP-1 expression significantly promoted FFA-induced apoptosis in DRP-1 WT (DRP-1 wild type) inducible INS-1-derived cell line, but not in DRP-1K38A (a dominant negative mutant of DRP-1) inducible INS-1-derived cell line. To validate these *in vitro* results, we transplanted DRP-1 WT or DRP-1 K38A cells into renal capsules of streptozotocin (STZ)-treated diabetic mice to study the apoptosis in xenografts. Consistent with the *in vitro* results, the over-expression of DRP-1 led to aggravated INS-1-derived cell apoptosis triggered by FFAs. In contrast, dominant-negative suppression of DRP-1 function as represented by DRP-1 K38A significantly prevented FFA-induced apoptosis in xenografts. It was further demonstrated that mitochondrial membrane potential decreased, while cytochrome c release, caspase-3 activation, and generation of reactive oxygen species (ROS) were enhanced by the induction of DRP-1WT, but prevented by DRP-1 K38A in INS-1-derived cells under FFA stimulation. These results indicated that DRP-1 mediates FFA-induced INS-1-derived cell apoptosis, suggesting that suppression of DRP-1 is a potentially useful therapeutic strategy for protecting against beta cell loss that leads to type 2 diabetes.

## Introduction

Type 2 diabetes (T2D) is associated with dyslipidemia, hyperglycemia, insulin resistance, and defects in insulin secretion from pancreatic beta cells [1]. It is also becoming clear that increased beta cell apoptosis is associated with diabetes in humans and animal models [2]–[5]. The exact prodiabetic events remain incompletely understood, but it has been hypothesized that the elevated levels of lipids, including increased free fatty acids (FFAs), in obese individuals may contribute to the pathophysiology of the disease [6]. Many studies have shown that chronic high levels of circulating FFAs were detrimental to beta cell function and survival [7]–[10]. Therefore, elucidating the molecular mechanisms underlying FFA-induced beta cell apoptosis would facilitate the understanding of T2D and open avenues for the development of new therapies [11].

Mitochondrial dysfunction has been implicated in FFA-induced beta cell apoptosis. However, molecular mechanisms linking mitochondrial dysfunction and FFA-induced beta cell apoptosis are not clear [12]–[14]. As a GTP-binding protein, dynamin-related protein 1 (DRP-1) is a mitochondrial fission protein whose expression promotes mitochondrial fragmentation. The expression of its dominant-negative form inhibits mitochondrial fission and thereby prevents apoptosis [15], [16]. Our previous studies found that hyperglycemia increased the expression of DRP-1 and yielded DRP-1-induced mitochondrial fission to cause mitochondrial fragmentation and apoptosis in INS-1-derived cells, while DRP-1 dominant-negative mutant impeded fission and apoptosis [17]. However, to our knowledge, the effects of DRP-1 on FFA-induced beta cell apoptosis have not been explored so far.

To clarify the possible involvement of DRP-1 in lipotoxicity-induced beta cell apoptosis, we first examined the effects of a high level of palmitate on the expression of DRP-1 and the apoptosis in INS-1 cells and rat islets. Two, previously established, stable INS-1-derived cell lines that can induce the expressions of wild-type DRP-1 (DRP-1 WT) and its dominant-negative mutant (DRP-1 K38A) were then used to investigate the role of DRP-1 on lipotoxicity-induced apoptosis *in vitro* and *in vivo*. In addition, the relevant mechanisms by which DRP-1 mediates INS-1-derived cell apoptosis were further analyzed through the assessment of various morphological and functional parameters.

## Results

### Apoptosis-inducing saturated FFA palmitate upregulated DRP-1 in INS-1 cells and rat islets

**Figure 1:**
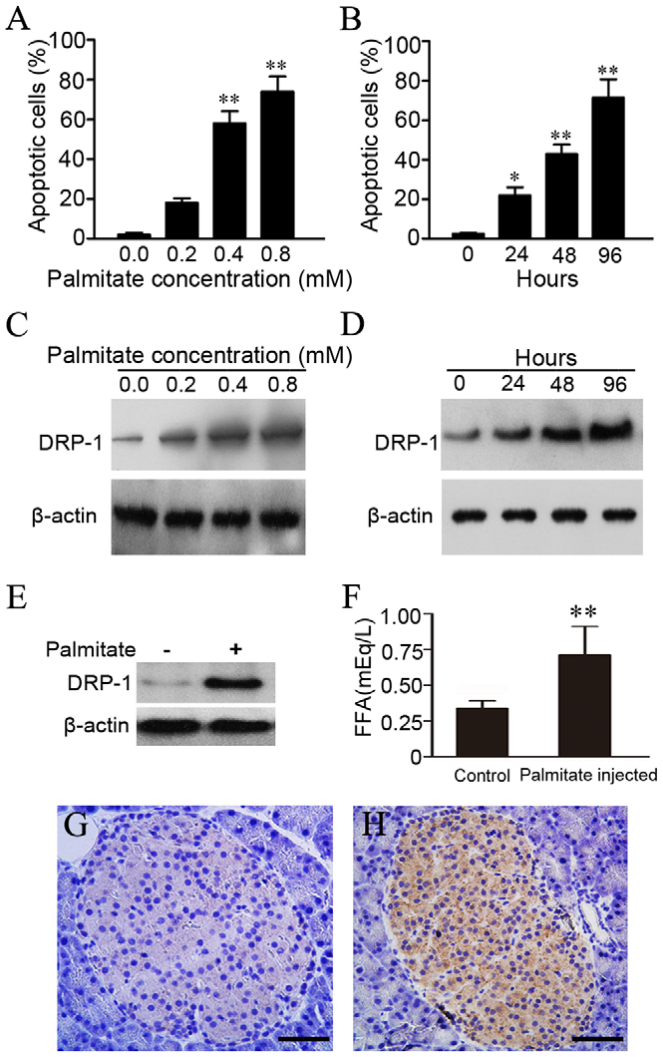
Apoptosis-inducing saturated FFA, palmitate upregulated DRP-1 in INS-1 cells and rat islets. (A) Dose-response and (B) time-course of palmitate-induced apoptosis in INS-1 cells. To study dose-response, cells were cultured with the indicated doses (0 mM, 0.2 mM, 0.4 mM, and 0.8 mM) of palmitate for 24 h. To study the time-course, cells were cultured in a medium containing 0.2 mM palmitate for the indicated amounts of time (0 h, 24 h, 48 h, and 96 h). Analysis of apoptosis in INS-1 cells was done by annexin V staining. The data are presented as mean percentage ± S.E. of annexin V-positive cells. The experiments were carried out for three independent times using triplicate samples (*p<0.05, **p<0.01, ANOVA/Tukey test). (C) Dose-response and (D) time-course of palmitate on DRP-1 expression in INS-1 cells. To study the dose-response, cells were cultured with the indicated doses of palmitate for 24 h. To study the time-course, cells were cultured in a medium containing 0.2 mM palmitate for the indicated amounts of time (E) DRP-1 was augmented in isolated islets treated with 0.5 mM palmitate for 24 h. (F) Serum FFA levels were increased after palmitate stimulation. C57BL/6 mice were i.p. injected with 0.5 ml of 5 mM palmitate once daily for 5 days. Their serum FFA levels 2 h after injection at the fifth day were determined utilizing the nonesterified fatty acid assay kit. Samples were tested in duplicate. Mean ± S.D. of the palmitate injected groups and control groups (BSA injected) are shown (n = 6 for each group). The difference is statistically significant (**p<0.01, unpaired two-tailed t test). (G, H) Increased FFA levels upregulated islet DRP-1 expression. After i.p. injection of palmitate once daily for 5 days, immunohistochemistry was performed to evaluated islet DRP-1 expression in (G)control group and (H) palmitate injected group (n = 6). Scale bar, 50 µm.

We initially examined the effects of saturated FFA palmitate on the apoptosis in the rat pancreatic beta cell line, INS-1. Cell apoptosis was analyzed by annexin V staining. We found that palmitate induced INS-1 cell apoptosis in time-dependent and dose-dependent manners (Fig. 1A and 1B), which was consistent with previous studies [18], [19]. To determine whether the apoptotic response to palmitate was associated with DRP-1 expression, we explored the effects of palmitate on DRP-1 expression in INS-1 cells and rat islets. INS-1 cells were treated with different concentrations of palmitate (0.1, 0.2, 0.4, and 0.8 mM) for different durations (12, 24, 36, and 48 h). As shown in Fig. 1C and 1D, DRP-1 expression increased as the duration and concentration of palmitate treatment of INS-1 cells increased. Chronic exposure of isolated rat pancreatic islets to palmitate resulted in an analogous increase in DRP-1 expression as observed in INS-1 cells (Fig. 1E). In addition, when palmitate was intraperitoneally (i.p.) injected into mice once daily for 5 days, serum FFA levels increased (Fig. 1F) accompanied by a significantly amplified expression of DRP-1 at day 5 (Fig. 1G and 1H). In short, DRP-1 was upregulated in beta cells upon stimulation by FFAs, and such an upregulation correlated with aggravated cell apoptosis.

### Characterization of DRP-1 WT and DRP-1 K38A inducible cell lines

**Figure 2:**
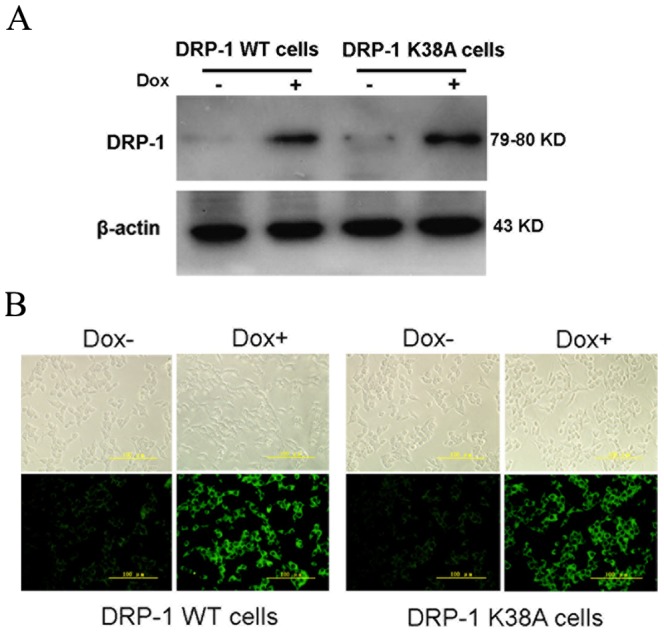
Characterization of the DRP-1WT and DRP-1 K38A inducible beta cell lines. (A) Western blot analysis for Dox-induced DRP-1 protein expression in DRP-1 WT and DRP-1 K38A cells. (B) DRP-1 WT and DRP-1 K38A proteins were induced by Dox. Phase-contrast images (top) and immunofluorescence staining (bottom). Scale bar, 100 µm.

In our previous work, we established two stable cell lines capable of an inducible expression of DRP-1 WT or dominant-negative mutant DRP-1 K38A [17]. In the present study, these two cell lines were used to investigate whether DRP-1 was involved in FFA-induced INS-1-derived cell apoptosis. The cells were induced with 500 ng/ml of doxycycline (Dox) for 48 h and the DRP-1 expression was analyzed by western blot (Fig. 2A) and immunofluorescence staining (Fig. 2B). As shown in Fig. 2, Dox markedly induced DRP-1 expression. However, Dox alone did not affect the endogenous DRP-1 expression in the INS-r9 cell control (data not shown).

### DRP-1 mediated FFA-induced INS-1-derived cell apoptosis *in vitro*

**Figure 3:**
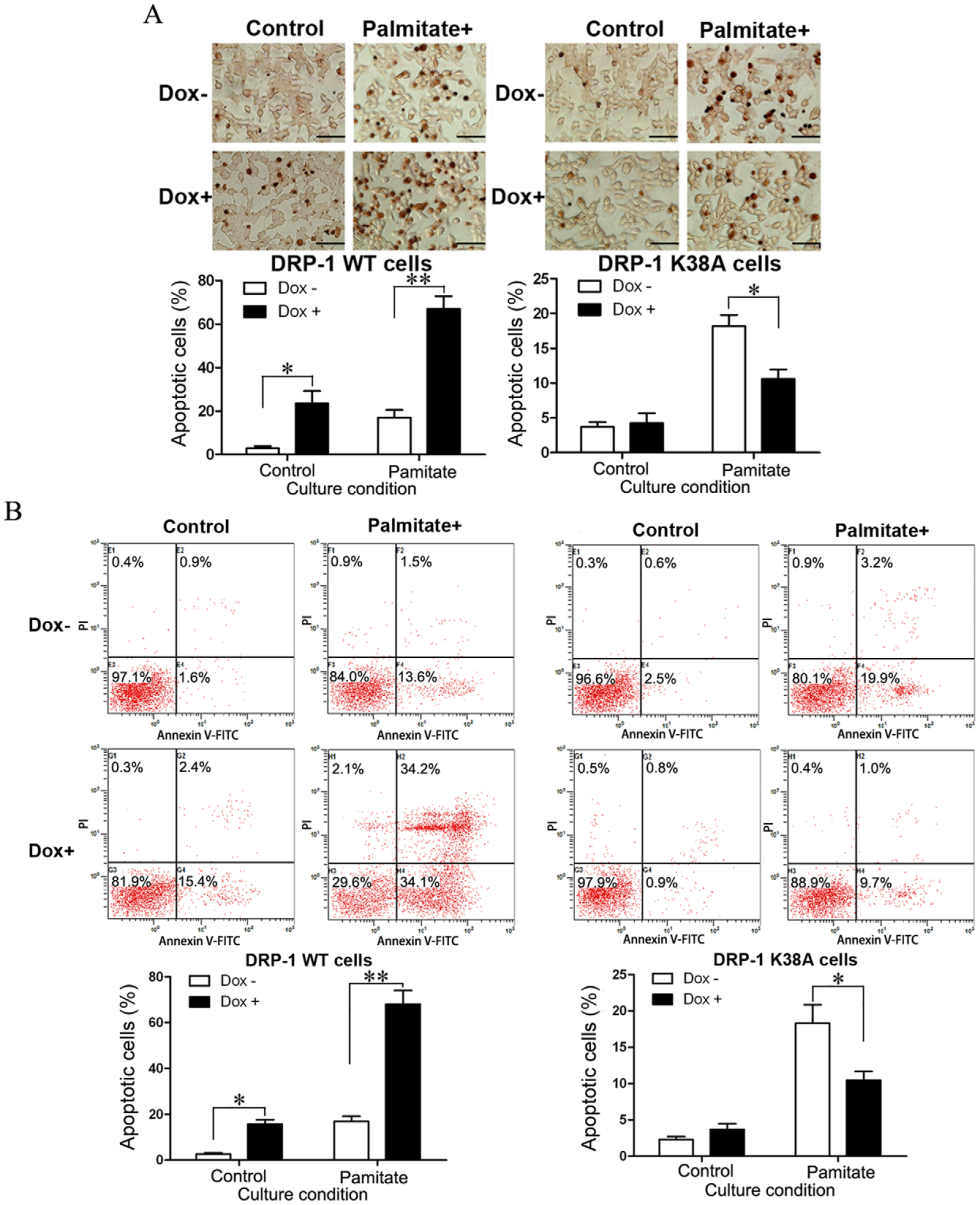
Analysis of apoptosis in DRP-1 WT and DRP-1 K38A cells. DRP-1 WT and DRP-1 K38A cells were cultured in the presence (black bars) or absence (white bars) of 500 ng/ml Dox for 48 h, and then treated with 0.2 mM palmitate for 24 h. (A) TUNEL staining of apoptosis in DRP-1 WT cells and DRP-1 K38A cells. Columns represent mean ± S.E. of three different experiments conducted in triplicate (*p<0.05, **p<0.01, ANOVA/Tukey test). Scale bar, 50 µm. (B) Flow cytometric analysis of apoptosis in DRP-1 WT cells and DRP-1 K38A cells. This assay could discriminate between intact cells (FITC−/PI−), early apoptotic cells (FITC+/PI−), late apoptotic cells (FITC+/PI+), and necrotic cells (FITC−/PI+). The bar graph shows the average apoptosis rate (early apoptosis and late apoptosis) of INS-1-derived cells with different Dox and palmitate treatments. Columns represent mean ± S.E. of three different experiments conducted in triplicate (*p<0.05, **p<0.01, ANOVA/Tukey test).

To investigate the possible involvement of DRP-1 in FFA-induced INS-1-derived cell apoptosis, DRP-1 WT or DRP-1 K38A cells were cultured in the presence or absence of 500 ng/ml Dox for 48 h and then treated with or without 0.2 mM palmitate for 24 h. The apoptosis in DRP-1 WT and DRP-1 K38A cells was assessed by terminal deoxynucleotidyl transferase-mediated dUTP nick-end labeling (TUNEL) staining (Fig. 3A) and annexin V-PI double staining (Fig. 3B). Induction of DRP-1 WT resulted in INS-1-derived cell apoptosis and it was significantly enhanced by the addition of palmitate. In contrast, DRP-1 K38A induction did not cause INS-1-derived cell apoptosis. Instead, it partially prevented FFA-induced apoptosis in INS-1-derived cells (Fig. 3). These results indicate that DRP-1 is involved in FFA-induced INS-1-derived cell apoptosis.

### DRP-1 mediated FFA-induced INS-1-derived cell apoptosis *in vivo*

**Figure 4:**
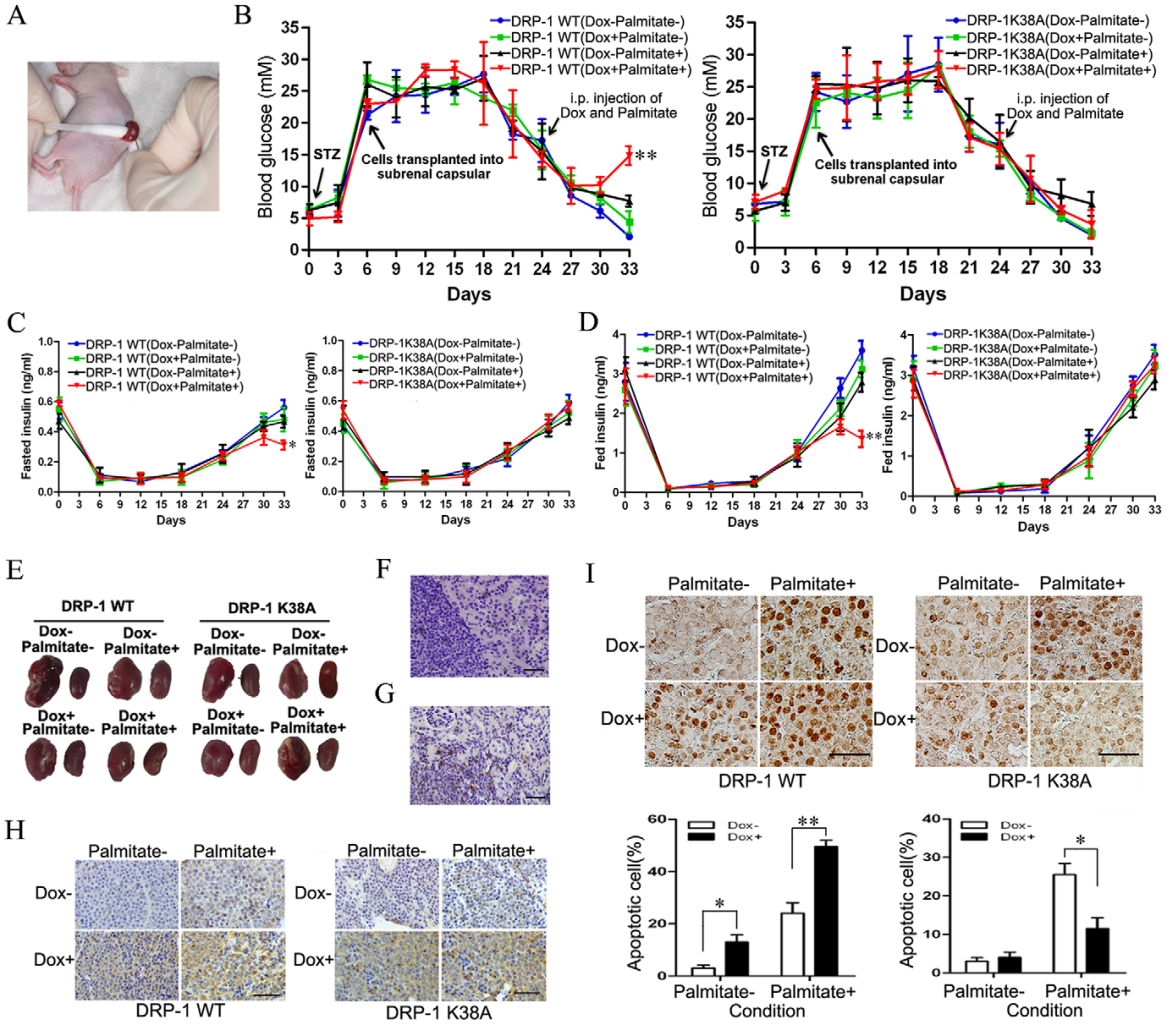
DRP-1 mediated FFA-induced INS-1-derived cell apoptosis *in vivo*. (A) Subrenal capsule implantation surgery. Left kidney of a nu/nu mouse was exposed. DRP-1 WT cells or DRP-1 K38A cells were injected under the capsule membrane. (B) Average blood glucose levels in fasting animals of the eight experimental groups. Data are expressed as mean ± S.E. (n = 6, **p<0.01, ANOVA/Tukey test). (C) Average blood insulin levels in fasting animals of the eight experimental groups. Data are expressed as mean ± S.E. (n = 6, *p<0.05, ANOVA/Tukey test). (D) Average blood insulin levels in fed animals of the eight experimental groups. Data are expressed as mean ± S.E. (n = 6, **p<0.01, ANOVA/Tukey test). (E) The mouse kidneys of each group at day 33. In each group, the left kidneys were transplanted with DRP-1 WT cells or DRP-1 K38A cells. (F) H&E staining showed DRP-1 WT cells can form tumors after subrenal capsules transplantation. DRP-1 K38A cells also can form tumors similar to DRP-1 WT cells (data not shown). (G) Example case of insulin expression in DRP-1 WT cell xenografts observed by immunohistochemical staining. Insulin is also expressed in DRP-1 K38A cell xenografts (data not shown). (H) Example case of DRP-1 expression in DRP-1 WT cell or DRP-1 K38A cell xenografts of each group observed by immunohistochemical staining. (I) The TUNEL staining was employed to detect the apoptosis in DRP-1 WT cell or DRP-1 K38A cell xenografts. Data are expressed as mean ± S.E. (n = 6, *p<0.05, **p<0.01, ANOVA/Tukey test). Scale bar, 50 µm.

To validate our *in vitro* results, we transplanted DRP-1 WT cells or DRP-1 K38A cells into the renal capsules of streptozotocin (STZ)-treated diabetic mice (Fig. 4A). Since the INS-1 cell line is derived from rat insulinoma, the two INS-1-derived cell lines would be xenografts in the renal capsules (Fig. 4E and 4F). After transplantation, the fasting blood glucose of the mice began to decline gradually (Fig. 4B). To interpret the blood glucose data, the corresponding insulin data of fed and fasting animals were also examined (Fig. 4C and 4D). These data suggested that INS-1-derived cells as xenografts indeed secreted insulin to lower blood glucose (Fig. 4G). At day 18 post-transplantation, the mice were i.p. injected once daily with palmitate to increase the plasma FFA concentration and Dox to induce DRP-1 WT or DRP-1 K38A expression (Fig. 4H). After 3 days of these treatments, plasma FFA increased significantly (data not shown). However, the fasting blood glucose of the mice still decreased gradually (Fig. 4B). After 9 days of these treatments, induction of DRP-1 WT in xenografts led to increased blood glucose (Fig. 4B) and decreased insulin secretion (Fig. 4C and 4D). However, induction of DRP-1 K38A had no such effects.

We speculated that different changes in blood glucose and insulin secretion were due to different effects of DRP-1 WT and DRP-1 K38A on cell apoptosis under high plasma FFA conditions. To validate our speculation, TUNEL staining was employed to detect the apoptosis in DRP-1 WT or DRP-1 K38A cell xenografts. Over-expression of DRP-1 WT enhanced FFA-induced apoptosis in the xenograft cells. As expected, DRP-1 K38A over-expression decreased FFA-induced apoptosis (Fig. 4I). It is likely that the different apoptosis rate in xenografts is one of the reasons for the changes in blood glucose and insulin secretion. Together, these data indicate that DRP-1 mediates FFA-induced INS-1-derived cell apoptosis *in vivo*.

### DRP-1 mediated FFA-induced apoptosis through the activation of the mitochondrial apoptosis pathway

**Figure 5:**
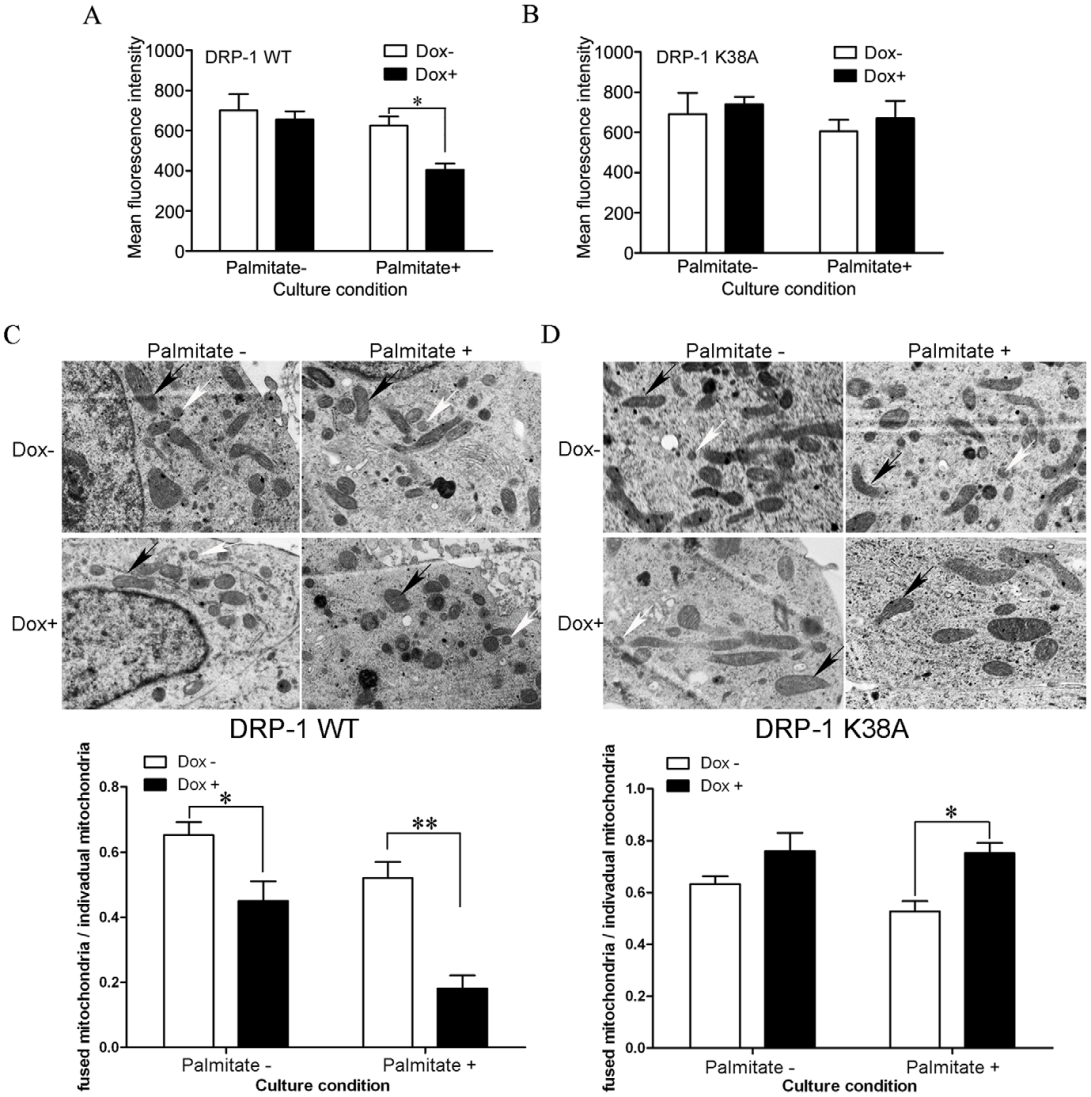
Effect of DRP-1 over-expression on mitochondrial membrane potential and mitochondrial morphology in DRP-1 WT cells and DRP-1 K38A cells. (A, B) Effects of (A) DRP-1 WT and (B) DRP-1 K38A over-expression on mitochondrial membrane potential. Data are presented as mean ± S.E. of three different experiments conducted in triplicate (*p<0.05, ANOVA/Tukey test). (C, D) Effects of DRP-1 WT and DRP-1 K38A over-expression on mitochondrial morphology. Transmission electron microscopy of mitochondrial morphology in (C) DRP-1WT cells and (D) DRP-1K38A cells in different palmitate and Dox conditions. Black arrows point to the typical fused mitochondria (elongated mitochondria). White arrows point to the typical punctiform mitochondria. The bar graph shows the average ratio of fused mitochondria vs. individual mitochondria (elongated & small punctiform mitochondria) for each of the experimental conditions. Data are presented as mean ± S.E. of three different experiments conducted in triplicate (*p<0.05, **p<0.01, ANOVA/Tukey test).

Since DRP-1 plays an important role in activating the mitochondrial apoptosis pathway, the mechanisms underlying DRP-1 involvement in FFA-induced INS-1-derived cell apoptosis were further investigated. The mitochondrial membrane potential was measured by flow cytometry with the potential-sensitive probe DiOC_6_ (3) [20]. As shown in Fig. 5, induction of DRP-1 WT led to mitochondrial membrane potential depolarization in INS-1-derived cells cultured under lipotoxic condition (Fig. 5A). In contrast, such effects were prevented in DRP-1 K38A cells (Fig. 5B). These data were consistent with reports that mitochondria eventually depolarized during the release of cytochrome c and activation of the caspase cascade [21]–[23].

**Figure 6:**
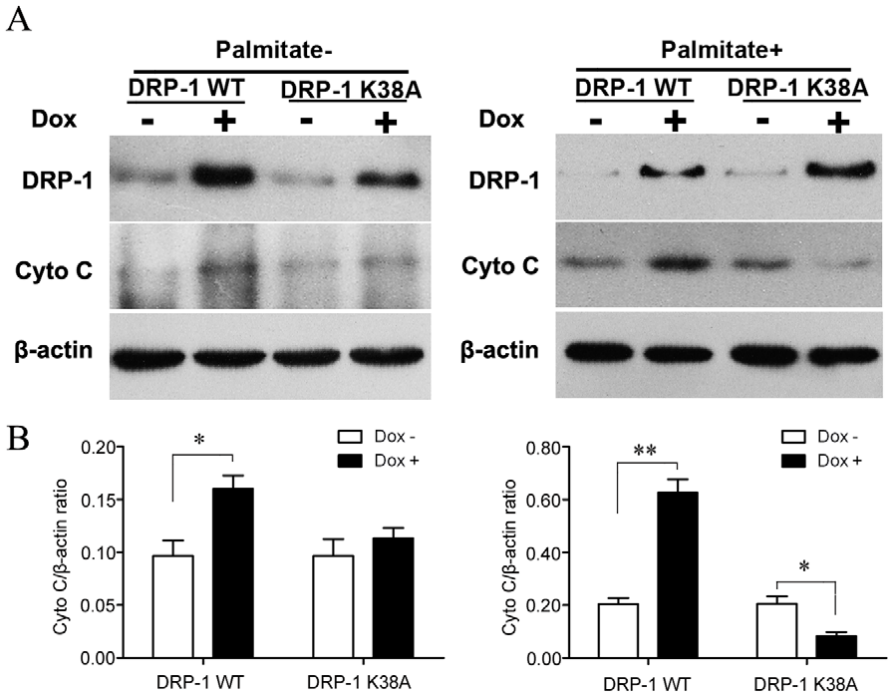
The effect of DRP-1 on cytochrome c release in INS-1-derived cells. (A) A representative western blot of DRP-1 and cytoplasmic cytochrome c expression in DRP-1 WT and DRP-1 K38A cells with different Dox and palmitate treatments. (B) A bar graph showing the relative cytochrome c release in INS-1-derived cells with different Dox and palmitate treaments. The relative intensity of each band was determined as a ratio to its corresponding beta-actin band. Data are presented as mean ± S.E. of three different experiments (*p<0.05, **p<0.01, ANOVA/Tukey test).

Transmission electron microscopy showed that palmitate caused morphological changes of mitochondria, from long into round and short shapes (Fig. 5C and 5D). This suggested mitochondrial fission. Induction of DRP-1 WT, which is known to cause mitochondrial fission, resulted in similar morphological changes of mitochondria (Fig. 5C). Moreover, mitochondrial fragmentation was even more pronounced when DRP-1 WT induction was combined with palmitate (Fig. 5C). In contrast, induction of DRP-1K38A abolished the effects of palmitate on mitochondrial fission in INS-1-derived cells (Fig. 5D). Furthermore, as demonstrated by western blot, induction of DRP-1 WT led to mitochondrial cytochrome c release under lipotoxic conditions. However, cytochrome c release was not observed in DRP-1 K38A cells under the same conditions (Fig. 6A, 6B and 6C). These results suggested that DRP-1 mediates FFA-induced apoptosis through cytochrome c release.

**Figure 7:**
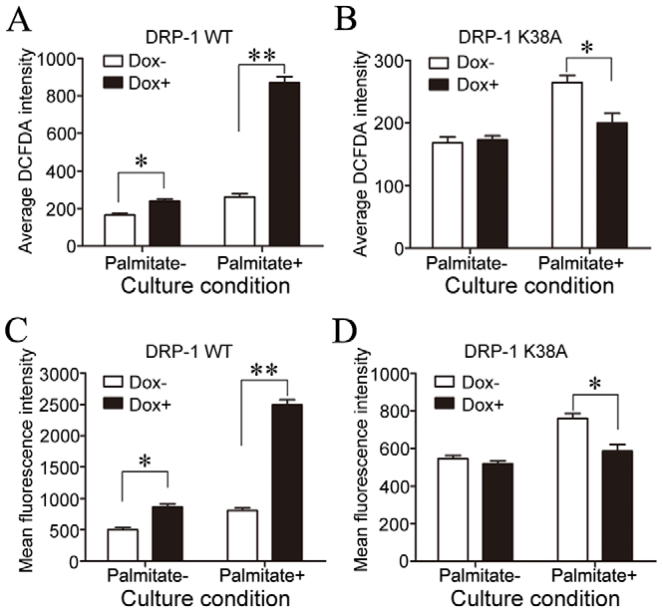
The effect of DRP-1 on ROS production and caspase-3 activity in DRP-1 WT and DRP-1 K38A cells. Data are presented as mean ± S.E. of three different experiments conducted in triplicate (A, B) The effect of DRP-1 on ROS production in DRP-1 WT and DRP-1 K38A cells (*p<0.05, **p<0.01, ANOVA/Tukey test). (C, D) The effect of DRP-1 expression on caspase-3 activity in DRP-1 WT cells and DRP-1 K38A cells (*p<0.05, **p<0.01, ANOVA/Tukey test).

To ascertain the involvement of reactive oxygen species (ROS) in DRP-1 induced apoptosis, the production of ROS was analyzed by flow cytometry in DRP-1 WT and DRP-1 K38A cells following FFAs addition. Flow cytometric analysis showed that induction of DRP-1 WT enhanced FFA-induced ROS production in INS-1-derived cells, while induction of DRP-1 K38A inhibited ROS generation in INS-1-derived cells under the same conditions (Fig. 7A and 7B).

To further assess whether activation of executioner caspase was involved in DRP-1 induced cell apoptosis, caspase-3 activity was measured by monitoring the cleavage of a fluorigenic caspase substrate in DRP-1 WT and DRP-1 K38A cells under FFA stimulation. These results showed that induction of DRP-1 WT enhanced FFA-induced caspase-3 activation, whereas induction of DRP-1 K38A prevented caspase-3 activation in INS-1-derived cells (Fig. 7C and 7D).

## Discussion

T2D is a complex disease that is accompanied by elevated levels of FFAs, which contribute to beta cell dysfunction and apoptosis, referred to as lipotoxicity. Emerging evidence point to the importance of mitochondrial dysfunction in FFA-induced beta cell apoptosis [13], [14], [24], [25]. However, molecular mechanisms linking mitochondrial dysfunction and FFA-induced beta cell apoptosis are not clear. Whether some protein factors directly participating in mitochondrial fragmentation have important roles in FFA-induced beta cell apoptosis has not been examined. The present study tested the hypothesis that DRP-1, a key mitochondrial fission modulator, has important roles in INS-1 beta cell apoptosis under lipotoxic conditions.

DRP-1 is a member of the dynamin superfamily of GTPases [26]. The level of *Drp-1* mRNAs is high in brain, moderate in skeletal and heart muscles, and low in pancreatic tissues. It has been shown that the expression of DRP-1 is required for mitochondrial fragmentation and programmed cell death of non-beta cells [27]. Furthermore, our recent study revealed that the expression of DRP-1 was upregulated in beta cells in response to high glucose, and DRP-1 mediated high glucose-induced INS-1 beta cell apoptosis [17]. However, whether DRP-1 is involved in FFA-induced INS-1 beta cell apoptosis should be further examined.

In the present study, DRP-1 was upregulated in rat islets and INS-1 beta cells under FFA stimulation and it correlated with increased cell apoptosis. We also found that the induction of DRP-1 expression significantly promoted FFA-induced apoptosis in the DRP-1 WT inducible beta cell line, but not in the DRP-1 K38A inducible beta cell line. These results confirmed our speculation that DRP-1 plays an important role in FFA-induced apoptosis in INS-1-derived cells. It also suggested that DRP-1 acts under a two-hit model, in which signaling event(s) (hit 1) derived from FFA stimulation as well as DRP-1 (hit 2) are both required to induce beta cell apoptosis. For normal islets, high DRP-1 expression (hit 2) could be a consequence of FFA (hit 1). We hypothesized that in individuals with abnormally high basal DRP-1 expression in islets, less of hit 1 might be sufficient to cause excessive islet damage. Thus, these individuals are more prone to T2D development when facing increased serum lipids. A further epidemiological study on T2D prevalence in individuals having enhanced DRP-1 expression will determine whether DRP-1 is a bona fide diabetic risk gene in humans.

To validate the *in vitro* results, we transplanted DRP-1 WT or DRP-1 K38A cells into the renal capsules of STZ-treated diabetic mice. After transplantation, mice were injected daily with FFA in the presence or absence of Dox to induce DRP-1 WT and DRP-1 K38A expression. Over-expression of DRP-1 WT enhanced FFA-induced apoptosis in the xenograft cells. As expected, DRP-1 K38A over-expression decreased FFA-induced apoptosis. This result provided direct evidence that DRP-1 mediates FFA-induced INS-1-derived cell apoptosis *in vivo*. Moreover, this subrenal capsular transplantation animal model offers a new option for studying gene function in INS-1 cells *in vivo*.

The mechanisms underlying DRP-1 involvement in FFA-induced apoptosis were further examined. It has been demonstrated that mitochondrial morphology and function are closely related. Under physiological conditions, mitochondrial size, morphology, and number are controlled by a balance of mitochondrial fission and fusion performed by an increasing set of “shaping” proteins that seem to have a regulatory role in the mitochondrial pathways of cell death. DRP-1 was reported to be a mitochondrial pro-fission factor [28]. During cell apoptosis, mitochondria undergo multiple changes that culminate in the release of cytochrome c and other pro-apoptotic factors. On the other hand, expression of pro-fusion proteins or inhibition of pro-fission molecules reduces cell apoptosis [29], suggesting a close relationship between mitochondrial fragmentation and cell apoptosis.

In the present study, the treatment of INS-1-derived cells with high FFAs led to marked morphological changes, including the breakdown of the semi-reticular mitochondrial network into small point-sized organelles, suggesting predominant fission events. Accordingly, over-expression of DRP-1 WT resulted in similar changes in mitochondrial morphology. In addition, the synergistic effects of DRP-1 WT and high FFA concentration were clearly demonstrated. In contrast, induction of DRP-1K38A preserved the elongated form of mitochondria, indicating that fusion events overcame FFA-evoked fission events. Thus, we propose that DRP-1 plays a predominant role in the regulation of high FFA concentration-induced mitochondrial fission and ensuing apoptosis in INS-1-derived cells.

The mitochondria-dependent process of cell apoptosis can be divided into three phases: initiation, progression, and execution [29]. During the first phase, particular pro-apoptotic cellular signals arise and begin to alter the membrane permeability of the mitochondria. During the progression phase, the mitochondrial membrane is permeabilized, allowing cytochrome c to the slowly move into the cytosol. The final execution phase concludes with the total discharge of cytochrome c and the activation of catabolic proteases needed for DNA laddering observed during cell apoptosis.

It has been shown that mitochondrial fragmentation is required for the release of apoptogenic factors from the mitochondrial inter-membrane space. One of the best characterized factors is cytochrome c. Once released into the cytosol, cytochrome c binds to Apaf-1 to form a complex with caspase-9 [30]. This large complex initiates the activation of downstream caspases. Induction of DRP-1K38A resulted in long, fused mitochondria by inhibiting mitochondrial fission, thereby leading to reduced cytochrome c release and cell apoptosis [31].

Mitochondrial fission is an early step of cell apoptosis, a process involving mitochondrial fragmentation, fission, and eventually disappearance. Mitochondrial fission required the activity of DRP-1, while mutation of its catalytic GTPase active site blocked mitochondrial fission [20]. It was reported that mitochondrial fission may encourage cell apoptosis by increasing the amount of outer membrane surface area available for pore formation. It has also been suggested that the outer membrane permeabilization is directly caused by the fission machinery, since the membrane continuity is essentially disrupted by the fission process. Mitochondrial fusion may protect cells from apoptosis by triggering the repair of damage to the outer membrane. Our results showed that, under high FFA conditions, over-expression of DRP-1 increased ROS generation, decreased mitochondrial membrane potential, promoted cytochrome c release, induced caspase-3 activation and induced apoptosis in INS-1-derived cells. In contrast, under the same conditions, induction of DRP-1K38A efficiently reduced mitochondrial fragmentation, ROS generation, and cell apoptosis. The morphological study demonstrated that mitochondrial fission events were predominant in induced DRP-1 WT cells, while mitochondrial fusion events were predominant in induced DRP-1K38A cells. These results suggested that DRP-1-induced mitochondrial fragmentation occurs when mitochondrial fission dominates over mitochondrial fusion. The high FFA-evoked mitochondrial fragmentation is in accordance with a previous report using other cell types [32].

In addition, it was reported that fission 1 (Fis1) and optic atrophy 1 (OPA1) mRNA levels were drastically upregulated in INS-1 cells upon FFA stimulation, suggesting other mitochondrial fission or fusion genes might also participate in high FFA-induced INS-1-derived cell apoptosis (data not shown). The effects of Fis1 or OPA1 on the high FFA-induced apoptosis in INS-1-derived cells are under investigation.

In summary, our results indicate that increased DRP-1 expression may explain FFA-induced beta cell apoptosis. DRP-1 may induce INS-1-derived cell apoptosis through mitochondrial fission, cytochrome c release, ROS generation and caspase-3 activation. In contrast, dominant-negative suppression of DRP-1 function by DRP-1 K38A largely prevented FFA-induced INS-1-derived cell apoptosis. Thus, for the first time, we suggest that DRP-1-mediated mitochondrial fission is an essential mechanism underlying FFA-induced INS-1-derived cell apoptosis. Inhibition of DRP-1 function could represent a novel approach for beta cell protection in T2D.

## Methods

### Ethics Statement

This study was carried out in strict accordance with the recommendations in the Guide for the Care and Use of Laboratory Animal of the National Institutes of Health. The protocol was approved by the Committee on the Ethics of Animal Experiments of China-Japan Friendship Hospital (Permit Number: 008#201109). All surgery was performed under sodium pentobarbital anesthesia, and all efforts were made to minimize suffering. The 8-week-old female nu/nu mice obtained from Jackson Laboratory (Vitalriver, Beijing, China) were maintained in a specific pathogen-free facility at the Experimental Center of China-Japan Friendship Hospital accredited for animal care by the Chinese Association for Accreditation of Laboratory Animal Care.

### Preparation of palmitate

Palmitate/bovine serum albumin (BSA) conjugates were made by soaping palmitate with NaOH and mixing with BSA [33]. In brief, a 20 mM solution of palmitate in 0.01 M NaOH was incubated for 30 min at 70°C. Then, 5% fatty acid-free BSA in phosphate-buffered saline (PBS) was added to the fatty acid soaps in a 3∶1 volume ratio. The resulting conjugates contained 5 mM palmitate and 3.75% BSA. For *in vitro* experiments, the palmitate/BSA conjugates were diluted in RPMI 1640 (11.1 mM glucose) supplemented with 10% fetal calf serum (FCS, Sigma-Aldrich Company, St. Louis, MO, USA). The concentration of BSA in 0.2 mM palmitate is 0.15%. For *in vivo* i.p. injection experiments, palmitate/BSA conjugates were not diluted. The mice were i.p. injected with 100 mg/kg palmitate per day.

### FFA Measurements

Blood samples were taken 2 h after the last palmitate injection and stored at −20°C until use. Their FFA concentrations were determined utilizing the nonesterified fatty acid assay kit (Wako Chemicals, Richmond, VA, USA) following a modified version of the manufacturer's protocol to accommodate a 96-well microplate [34].

### Immunohistochemistry

Rat pancreases were excised, fixed in 4% paraformaldehyde, and processed for paraffin embedding. Pancreatic tissue sections (5 µm) were immunostained with mouse anti-DRP-1 (BD Transduction Laboratories, Lexington, KY, USA; 1∶100 dilution), biotinylated secondary antibodies and horseradish peroxidase-conjugated antibiotin using an ABC-Peroxidase kit (Vector Laboratories, Burlingame, CA, USA) according to the manufacturer's protocols.

### Establishment of INS-1 stable cell lines capable of inducible expressions of DRP-1WT and DRP-1 K38A

Rat insulinoma INS-1 cell-derived clones were cultured in RPMI 1640 supplemented with 10% fetal calf serum, 50 µM 2-mercaptoethanol and 11 mM glucose [35]. The first step that establishes a stable clone INS-r9 cell line, which carries the reverse Dox-dependent transactivator [36], was described previously [37], [38]. The plasmid for second stable transfection was constructed by subcloning cDNA encoding rat *Drp-1* or its dominant-negative mutant *Drp-1 K38A* into the expression vector PUHD10-3 [36]. Within the dominant-negative mutant Drp-1 K38A, the critical lysine in the G1 consensus motif of the GTPase domain is changed into an alanine, which seemingly prevents GTP binding of DRP-1. This mutation was used in a previous study of mammalian DRP-1 [39]. The procedures for stable transfection, clone selection, and screening were previously described [37]. The DRP-1 WT or DRP-1 K38A cells were induced with 500 ng/ml of Dox for 48 h and the DRP-1 expression was analyzed by western blot and immunofluorescence as described previously [17], [40].

### Cell treament and apoptosis assay

For the TUNEL assay, DRP-1 WT cells or DRP-1 K38A cells were seeded in 96-well plates (4×104 cells per well) and treated with or without 500 ng/ml Dox for 48 h. Then, the cells were incubated with 0.2 mM palmitate or 0.15% BSA (control) in RPMI 1640 containing 10% FCS for 24 h. The cells were then washed, fixed in 4% paraformaldehyde, and permeabilized with 0.1% Triton X-100 in PBS/BSA solution. The TUNEL assay [41] was performed using in situ cell death detection kits (Roche, Indianapolis, IN, USA).

For annexin V staining, DRP-1 WT cells or DRP-1 K38A cells were seeded in 6-well plates (1×106 cells per well) and cultured for 48 h in the presence or absence of 500 ng/ml Dox. The cells were then incubated with 0.2 mM palmitate or 0.15% BSA in RPMI 1640 containing 10% FCS for 24 h. The cells were incubated with 10 µl annexin V-FITC and 5 µl (50 µg/ml) PI on ice and then analyzed by FACScan (Beckman Counter Epics XL, Miami, FL, USA). This assay was used to discriminate between intact cells (FITC−/PI−), early apoptotic cells (FITC+/PI−), late apoptotic cells (FITC+/PI+), and necrotic cells (FITC−/PI+).

### Assessment of mitochondrial membrane potential, caspase-3 activity, and ROS production

Measurement of mitochondrial membrane potential was accomplished using cells incubated with 10 nM DiOC6(3) for 5 min at 37°C. After incubation, the cells were washed one time and resuspended in PBS for flow cytometry. As a positive control for dissipation of mitochondrial membrane potential, we used cells treated with 10 µM of the mitochondrial membrane uncoupler carbonylcyanidem-chlorophenyl hydrazone (mCCP) at 37°C for 20 min.

Caspase-3 activity was measured by caspase-3 assay kit (Molecular Probes, Invitrogen, Carlsbad, CA, USA) following the manufacturer's protocol on harvested cells that were washed three times with PBS. A microplate fluorescence reader (Molecular Devices Corp, Sunnyvale, CA, USA) was used for detection.

ROS formation was analyzed by flow cytometry according to the method previously described [42]. After washing the cells of interest twice with PBS, we stained them with CM-H2 DCFDA (1 mM) in PBS (pH 7.2) at 37°C for 5 min and then counterstained with PI (to 10 mg/ml) for 1 min. We then performed flow cytometry (Becton Dickinson, San Jose, CA, USA) using excitation wavelength of 488 nm and emission wavelength of 535 nm.

### Detection of cytochrome c release

Detection of mitochondrial cytochrome c release was performed as described previously [43]. Briefly, the cells were harvested and suspended in buffer A (20 mM HEPES-KOH, pH 7.5, 10 mM KCl, 1.5 mM MgCl_2_, 1 mM EDTA, 1 mM EGTA, 1 mM dithiothreitol, 250 mM sucrose, and 1 mM phenylmethylsulfonyl fluoride) and lysed by a sonicator. The lysate was centrifuged at 1000×g for 10 min at 4°C. The supernatant was collected for further centrifugation at 13,000×g for 20 min. The protein content in the supernatant was determined by micro BCA protein assay kit (Biyuntian, Beijing, China). The cytosolic proteins (supernatant fractions) were separated by 15% SDS-PAGE and analyzed by western blotting with a polyclonal antibody against cytochrome c (Santa Cruz Biotechnology, Santa Cruz, CA, USA). Cytochrome c release was also detected by confocal microscopy. Briefly, the cells were washed once in PBS and incubated with 1 µM MitoTracker Red at 37°C for 15 min and then fixed with 4% formaldehyde for 15 min at room temperature. The cells were also stained with anti-cytochrome c antibody and FITC-conjugated antibody as previously described [44]. The cells were viewed under a confocal microscope (Olympus IX81, Tokyo, Japan). Multitrack scanning mode was used to record the double-labeled cells.

### Electron microscopy

The cells were fixed with 2.5% (v/v) glutaraldehyde for 30 min, dehydrated, embedded, sectioned and visualized under a JEM-1010 transmission electron microscopy (JEOL, Tokyo, Japan).

### Subrenal capsular animal model and TUNEL staining

We first used a relatively high single dose (180 mg/kg) of STZ (Sigma-Aldrich Company, St. Louis, MO, USA) for diabetic induction to ensure that the mouse pancreatic beta cells were irreversibly destroyed. At day 6, the subrenal capsular animal model was established in STZ-treated diabetic mice by transplanting 5×106 DRP-1 WT or DRP-1 K38A cells into the left kidney. At day 18 post-transplantation, the blood glucose of mice began to decline significantly. The mice were i.p. injected once daily with or without palmitate (100 mg/kg per day) and Dox (5 mg/kg per day) for 9 days. All mice were killed at day 33. The left kidneys were immediately fixed in 10% buffer formalin at room temperature overnight and subsequently embedded in paraffin. Sections from these blocks were then subjected to hematoxylin & eosin (H&E) staining, immunohistochemistry and TUNEL staining.

### Statistics

The data were presented as means ± S.E. Multiple comparisons between groups were performed using ANOVA followed by Tukey's post hoc test. Unpaired two-tailed t tests were used when the differences between two groups were analyzed. *P*<0.05 was considered statistically significant.
