# Correction: Involvement of Dynamin-Related Protein 1 in Free Fatty Acid-Induced INS-1-Derived Cell Apoptosis

**DOI:** 10.1371/annotation/831aa38a-a30b-4d47-a4b3-10f5b0709158

**Published:** 2013-08-07

**Authors:** Liang Peng, Xiuli Men, Wenjian Zhang, Haiyan Wang, Shiqing Xu, Qing Fang, Honglin Liu, Wenying Yang, Jinning Lou

A grant and its number were incorrectly omitted from the Funding statement. The grant and number are: National Science and Technology Major Project (No.2011ZX09102-010-03). 

