# Correction: Involvement of Dynamin-Related Protein 1 in Free Fatty Acid-Induced INS-1-Derived Cell Apoptosis

**DOI:** 10.1371/annotation/f8ab435d-accb-4383-8784-23e6edf82f56

**Published:** 2013-09-09

**Authors:** Liang Peng, Xiuli Men, Wenjian Zhang, Haiyan Wang, Shiqing Xu, Qing Fang, Honglin Liu, Wenying Yang, Jinning Lou

The number of the first-listed grant from the National Natural Science Foundation of China is incorrect. The correct grant number is: 81200616. 

